# DAILY STRESSOR APPRAISALS AND SELF-VIEWS OF AGING PREDICT SAME-DAY AFFECTIVE RATINGS

**DOI:** 10.1093/geroni/igac059.501

**Published:** 2022-12-20

**Authors:** Erica O'Brien, Shevaun Neupert

**Affiliations:** Pennsylvania State University, University Park, Pennsylvania, United States; North Carolina State University, Raleigh, North Carolina, United States

## Abstract

Prior work suggests that more frequent or higher exposure to stressors relates to less positive affect and more negative affect in daily life. Limited knowledge exists about whether subjective appraisals of such stressors (i.e., perceived negative impacts on daily routine, personal health and safety, and finances) also have negative links to daily well-being. This study examines this link using data from an 8-day daily dairy study (n=675 days) in an online sample of older adults (n = 110 people, ages 60-90). We also explored potential psychological moderators particularly relevant to the experience of aging (i.e., self-views of aging, S-VOA). Results from multilevel models indicate that people reported more negative affect and less positive affect on days with more negative appraisals, especially on those days when they also had more negative self-views of aging. These findings highlight S-VOA as psychological resources that help people cope with stressful events in everyday life.

